# Unusual regulation of the CO_2_ concentrating mechanism of marine chemolithoautotroph *Thiomicrospira pelophila*

**DOI:** 10.1128/aem.01529-25

**Published:** 2025-10-20

**Authors:** Jana Wieschollek, Ren R. Payne, Carlos Abel Morales Alvarez, Nick Cisneros, Holly David, Christopher Dixon, Hannah Grzech, Sarah Oster, Charles Kasban, Connor Lunsford, Jacqueline Mikhaylov, Nicole Nauman, Clare L. Dennison, Dale Chaput, Kathleen Scott

**Affiliations:** 1 Integrative Biology Department, University of South Florida 123421 , Tampa, Florida, USA; Georgia Institute of Technology, Atlanta, Georgia, USA

**Keywords:** CO_2_-concentrating mechanism, autotroph, carbon fixation, chemolithoautotroph

## Abstract

Autotrophic organisms fix dissolved inorganic carbon (DIC: CO_2_, HCO_3_^-^, CO_3_^-2^) into biomass, introducing organic carbon into the global carbon cycle. Many lineages of autotrophs developed CO_2_ concentrating mechanisms (CCMs) to grapple with the catalytic constraints of the carboxylase of the Calvin-Benson-Bassham cycle, ribulose-1,5-bisphosphate carboxylase/oxygenase (RubisCO). Carboxysomes and DIC transporters act together to form CCMs, which facilitate DIC fixation by autotrophic bacteria when DIC is scarce. Autotroph *Thiomicrospira pelophila* has carboxysomes, with an unusual carbonic anhydrase, and encodes six potential DIC-transporters, more than other chemolithoautotrophs. We conducted experiments on how these carboxysomes and multiple transporters are integrated into a functioning CCM in *T. pelophila*. *T. pelophila* expresses a functional CCM. Four out of six transporters were capable of DIC uptake when expressed in *E. coli*. However, only one transporter was upregulated under DIC limitation. Transcript abundances from carboxysome-related genes in *T. pelophila* did not differ under DIC limitation, and carboxysome abundance in DIC-limited cells did not increase as markedly as in other species. This abundance of transporter genes and absence of dramatic changes in carboxysome abundance distinguish the CCM of *T. pelophila* from those studied in other organisms.

**IMPORTANCE:**

Although the general composition of CCMs is conserved (carboxysomes and DIC transporters), the evolutionary origins of these components can differ (e.g., different lineages of carbonic anhydrase enzymes and transporters). Here, we show a new pattern of gene regulation in response to DIC limitation, suggesting an added level of diversity in CCM operation. Understanding these layers of diversity is key to discerning how these organisms function *in situ,* as well as how they or their CCM components could be engineered into organisms of industrial or agricultural importance.

## INTRODUCTION

All bacteria that rely on the Calvin-Benson-Bassham cycle face two problems: they must respond to changes in concentrations of environmental dissolved inorganic carbon (DIC: CO_2_, HCO_3_^-^, CO_3_^−2^), and also need to cope with enzymatic constraints. Environmental DIC availability can vary greatly, ranging from micromoles to millimolar (1–3). Additionally, at pH 7 and above, HCO_3_^-^ is the primary form of available DIC (4). RubisCO (ribulose 1,5-bisphosphate carboxylase/oxygenase), the Calvin-Benson-Bassham cycle carboxylase, can only utilize CO_2_ as substrate, not HCO_3_^-^ (5). RubisCO can also use O_2_ as a substrate for a wasteful side reaction (6). As a result, many lineages of autotrophs developed CCMs to contend with the catalytic constraints of RubisCO.

CCMs have been well studied in *Cyanobacteria* (7, 8) and chemolithoautotrophic *Gammaproteobacteria*, namely *Hydrogenovibrio crunogenus* and *Halothiobacillus neapolitanus* (9–12). These CCMs consist of two parts: 1) DIC transporters, which facilitate active transport of dissolved inorganic carbon, generating high levels of intracellular HCO_3_^-^, and 2) carboxysomes, which are cytoplasmic proteinaceous microcompartments that encapsulate RubisCO and carbonic anhydrase (CA) (13, 14). Cytoplasmic HCO_3_^-^ enters carboxysomes through selective pores in the carboxysome shell (15). Within the carboxysome, CA converts HCO_3_^-^ to CO_2_, which is fixed by RubisCO.

Though the overall function of the two components of CCMs in *Bacteria* appears to be uniform, there is substantial variety among transporters and enzymes. DIC transporters are quite diverse, including three evolutionarily independent forms in *Cyanobacteria*. Sodium-HCO_3_^-^ symporters SbtA (16) and BicA (a member of the SulP transporter family) (17) rely on membrane potential for transport. ABC transporter BCT1 (CmpABCD) hydrolyzes ATP for HCO_3_^-^ transport (18). HCO_3_^-^-transporting members of the SulP and SbtA families of transporters are also found in autotrophic *Pseudomonadota* (11, 19)*,* in addition to a new class of multi-subunit membrane spanning DIC-accumulating complexes (DAC [20]). Organisms with CCMs often carry genes for more than one DIC transporter, based on habitats. For those organisms with more than one DIC transporter, genes are differentially regulated based on their affinities for DIC (21).

Carboxysomes are also quite diverse. While all carboxysomes consist of a protein shell containing RubisCO and CA, there are two types of carboxysomes that can be distinguished, depending on the type of RubisCO they encapsulate. RubisCO enzymes are all evolutionarily related; those present in *Bacteria* include form I, which has eight large and small subunits (CbbL, CbbS) and form II, which only has large subunits (CbbM) (22). Form I RubisCO can be further subdivided into forms A-D (23). α-Carboxysomes, present in autotrophic *Pseudomonadota*, as well as *Cyanobacteria* from genera *Synechococcus* (marine members) and *Prochlorococcus,* carry form IA RubisCO (24), while β-carboxysomes, present in other *Cyanobacteria*, carry form IB RubisCO (25, 26).

Carboxysomal CAs also vary among carboxysomes. CA enzymes are quite diverse, with eight evolutionary independent forms currently known: α, β, γ, δ, ζ, η, θ, ι (27). β-carboxysomes can either contain a β-class or γ-class CA, or both (28). It was thought that all α-carboxysomes contain a β-class CA (CsoSCA [14]), though recently ι-CA was identified in carboxysomes in members of the genus *Thiomicrospira* (29).

The presence of non-carboxysomal RubisCO adds yet another layer of diversity to CCMs. While *Cyanobacteria* only have genes encoding carboxysomal RubisCO, members of *Pseudomonadota* often have genes encoding both carboxysomal (form I) and noncarboxysomal RubisCO (form I, form II [30]). Typically, *cbbM* is upregulated when CO_2_ is abundant, and carboxysomal *cbbL* and *cbbS* are upregulated under low CO_2_ conditions (31, 32).

Members of *Thiomicrospira* have an unusual CCM. Their carboxysomes carry ιCA instead of CsoSCA (29). Additionally, unlike other chemolithoautotrophs, the amount of RubisCO in *T. pelophila* does not increase under DIC limitation (33). Further, the genomes of members of *Thiomicrospira* encode more DIC transporters (3–5) than sister taxa *Thiomicrorhabdus* and *Hydrogenovibrio* (1–3 [19]). Given that members of this genus are found in a variety of habitats, including marine sediments (34, 35), soda lakes (36), soap lakes (37), and hypersaline lakes (38), they may have CCMs with unusual adaptations to these environments. The purpose of this work was to study the CCM in *Thiomicrospira pelophila* to clarify how the components and regulation of its CCM are regulated and function under DIC limitation.

## MATERIALS AND METHODS

### Cultivation of *T. pelophila* in chemostats

**Fig 1:**
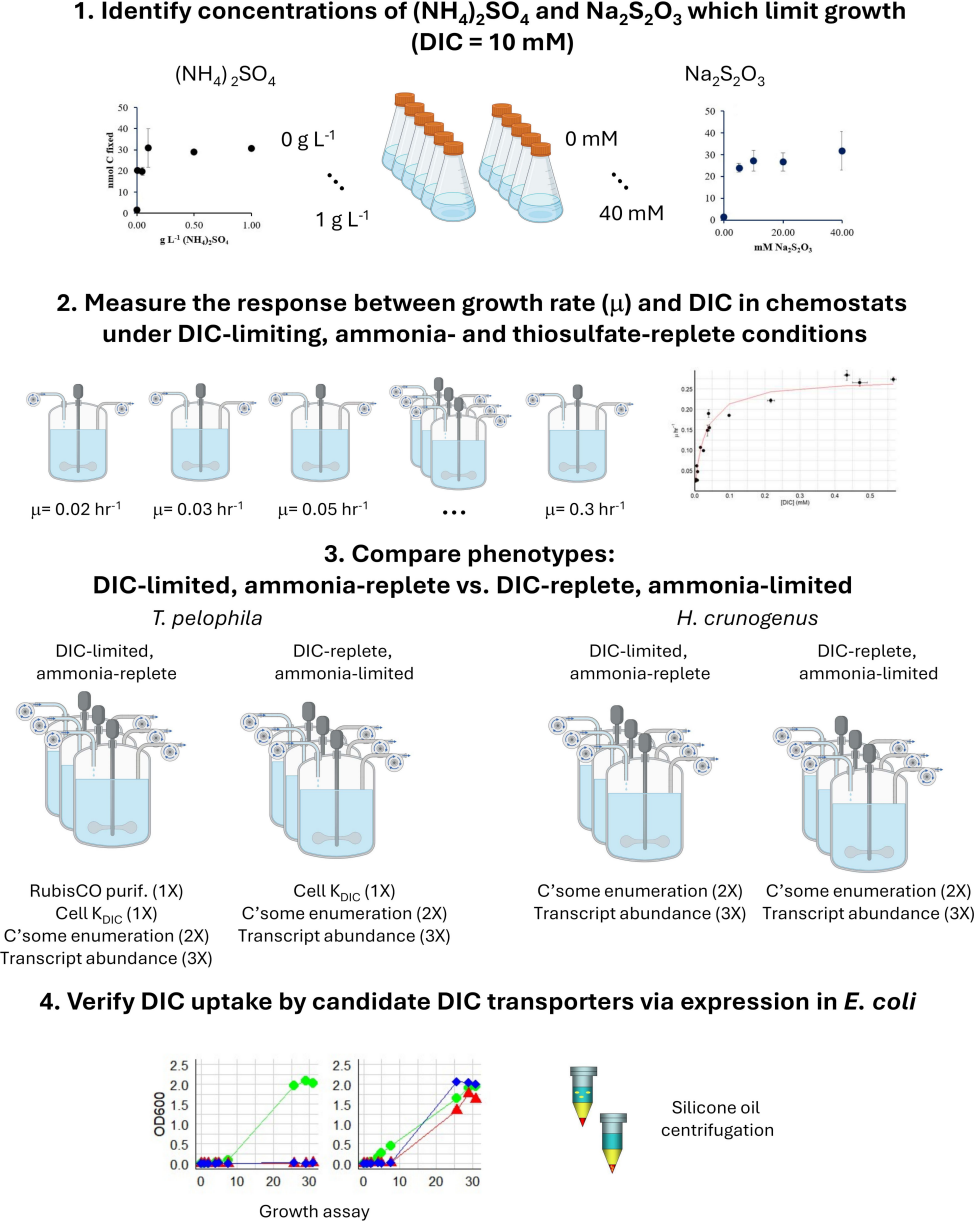
Study overview. In step 1, growth media were tested to determine the concentrations of ammonia or thiosulfate that would limit growth. For subsequent growth in chemostats, growth media were thiosulfate-replete (40 mM). In step 2, cells were grown in 15 DIC-limited, ammonia- and thiosulfate-replete chemostats, each with a set growth rate constant (µ), to elucidate the relationship between µ and the concentration of DIC. The results of step 2 were used to choose conditions under which *T. pelophila* was likely to express its CCM (low DIC concentrations capable of supporting growth). In step 3, phenotypes of *T. pelophila* cells were characterized when cultivated under DIC-limited, ammonia-replete vs. DIC-replete, ammonia-limited conditions. To verify that *T. pelophila’s* genome encodes DIC transporters necessary for CCM function, in step 4, candidate transporters were expressed in *E. coli* for characterization via growth assays and silicone oil centrifugation. Image created in BioRender. Scott, K. (2025) https://BioRender.com/tkzf3yy.

*T. pelophila* DSM 1534^T^ was obtained from the German Collection of Microorganisms and Cell Cultures and initially cultivated at 20°C under ambient air in thiosulfate-supplemented artificial seawater medium (TASW, pH 7.5, 1 µg L^−1^ vitamin B12 [12, 39]). Pilot experiments were undertaken to determine the best pH and O_2_ concentration for growth, as well as conditions under which ammonia or thiosulfate could be limiting (Fig. 1; Fig. S1). To establish the response of growth rate constant (μ) to DIC concentration, *T. pelophila* was grown at 20°C under DIC limitation in chemostats (Eppendorf, BioFlo 120; Fig. S2) in a modified TASW medium [TP medium, modified from reference 12: per liter, 25 g NaCl, 1.5 g MgSO_4_ × 7H_2_O, 0.3 g CaCl_2_ × 2H_2_O, 0.5 g K_2_HPO_4_, 25 g 3-(N-morpholino)propanesulfonic acid (MOPS) pH 7.0, 0.84 g NaHCO_3_, 5 µg vitamin B12, 1 mL SL-8 trace salts solution (SL-8 from reference 12)]. DIC in the growth medium reservoir was adjusted to 0.5 mM to provide sufficient biomass while still limiting growth. Oxygen concentrations were maintained at 80–100% air-saturated levels by monitoring culture O_2_ concentrations with O_2_ electrodes and periodically sparging with O_2_ gas. DIC concentrations in the growth medium reservoir and growth chamber were verified via gas chromatography as described previously (12, 40). The pH was monitored via pH electrodes and maintained between pH 7 and 7.5 by adding 10 N KOH. Chemostats reached steady state, based on constant optical densities (OD_440_ 0.1–0.2) and constant DIC in the growth chamber (Fig. S2) after 5L of growth medium had passed through the 1 L growth chamber. Once steady state had been reached, [DIC] and dilution rates (*D* = L of medium passing through the growth chamber ÷ growth chamber volume [1 L] = µ [41]) were measured in triplicate. In total, 15 chemostats, each set at a different constant dilution rate (= µ), were run (Fig. 1). The response of μ to [DIC] in the chemostat growth chamber was modeled with the Monod equation {μ = (μ_max_ × [DIC])/(K_DIC_ + [DIC]}, where [DIC] is the concentration of DIC in the growth chamber (42).

### Parameters for DIC fixation by cells and purified RubisCO

*T. pelophila* cells were grown in chemostats either under DIC or NH_3_ limitation with μ = 0.05 hr^−1^ to determine if whole-cell DIC fixation differed (Fig. 1). Cells were cultivated under DIC-limiting (NH_3_-replete), or NH_3_-limiting (DIC-replete) conditions (43), with the following modifications: for DIC-limited cells, TP medium in the growth medium reservoir (Fig. S2) consisted of 0.5 mM DIC, 7.6 mM (NH_3_)_2_SO_4_, and 100 mM MOPS. When μ was 0.05 hr^-1^, the concentration of DIC in the growth chamber was ~0.01 mM. For NH_3_-limited cells, the concentration of DIC in the growth medium reservoir was 5 mM, while the concentration of (NH_3_)_2_SO_4_ was 0.76 mM. To maintain high DIC concentrations in the growth chamber (4.3–4.5 mM; Fig. S2), NH_3_-limited culture growth chambers were sparged with 5% CO_2_, 95% O_2_ (vol/vol) instead of 100% O_2_. Cells were harvested from DIC- and NH_3_-limited chemostats via centrifugation (10,000 × *g*, 10 min, 4°C, Sorvall SLA-1500 rotor) and resuspended in fresh growth medium. K_DIC_ and V_max_ were determined by measuring DI^14^C fixation rates at a range of [DIC]. DI^14^C fixation rates were normalized to the amount of protein present in the assay (quantified via RC DC protein assay; BioRad Inc.). The response of carbon fixation rates (*v*) to [DIC] was modeled with the Michaelis-Menten equation {*v* = (V_max_ × [DIC]) / (K_DIC_ + [DIC])} (see “Statistical analyses,” below).

RubisCO was purified for K_CO2_ and V_max_ determination. Cells were harvested from a 5L DIC-limited chemostat (Fig. 1), and carboxysomes were purified as in (29). This carboxysome preparation was divided into aliquots and stored at −80°C. Three measurements of RubisCO kinetic parameters were undertaken. For each one, RubisCO was purified from an aliquot of the carboxysome preparation via three freeze-thaw cycles and centrifugation to rupture and remove carboxysome shells (44). RubisCO purity was verified by SDS-PAGE (45). RubisCO K_DIC_ and V_max_ values were determined by measuring rates of DI^14^C fixation at a range of [DI^14^C], as described in reference 46. DI^14^C fixation rates were normalized for protein concentrations and used to estimate K_DIC_ and V_max_ values as described above for whole-cell DI^14^C fixation. K_CO2_ values from DIC-limited chemostats, DIC-limited cells, NH_3_-limited cells, and RubisCO were calculated from K_DIC_ values using apparent pK_a_ values adjusted for buffer ionic strength as in reference 47.

### Enumeration of carboxysomes via transmission electron microscopy

To determine whether carboxysome abundance differed for cells grown under DIC limitation versus NH_3_ limitation, carboxysomes were visualized via transmission electron microscopy. *T. pelophila* was cultivated in TP medium in chemostats either under DIC or NH_3_ limitation as described above (Fig. 1). For comparison, *H. crunogenus* was also grown in TP medium, under the same conditions as *T. pelophila. H. crunogenus* was chosen as a reference organism, since its CCM is well-characterized (12). Four chemostats were grown per species, two DIC-limited and two NH_3_-limited.    

Samples were prepared and stained for electron microscopy as described in reference 31. Electron micrographs were taken of each sample, and carboxysomes were counted for all samples per square micrometer of cell cross-sectional area (31). Carboxysomes were counted from 100 cells from each chemostat. To avoid biasing the counts, the researcher counting the carboxysomes did not know the species or growth conditions corresponding to each image.

### Measuring differences in transcript abundance of CCM-associated genes in *T. pelophila* under DIC vs. NH_3_ limitation

To identify differences in transcript abundance under DIC-limited versus NH_3_-limited conditions, *T. pelophila* cells were cultivated as described above in three DIC-limited and three NH_3_-limited chemostats (Fig. 1). Cells were harvested and RNA was extracted as in (43). Transcripts of target genes were amplified for CCM-related genes using both previously published (19, 43) and new (Table S1) primers. QRT-PCR assays were carried out in the Applied Biosystems Step One real-time PCR system, using the QuantiTect SYBR Green RT-PCR kit (Qiagen, Inc.) and thermocycling parameters from reference 43. Relative amounts of transcripts were calculated using the 2^-ΔΔCT^ method (48), in which the gene encoding citrate synthase was used as a calibrator.

### Heterologous expression of potential DIC-transporter genes in *E. coli* Lemo21(DE3) *ΔyadF ΔcynT*

To determine whether potential DIC transporters from *T. pelophila* were capable of DIC transport, genes encoding them were cloned into the vector pBAD202 (Invitrogen, Inc.), in which their expression was regulated by *araBp*, the promoter from the *araBAD* operon (Fig. 1). The Department of Energy Joint Genome Institute synthesized pBAD202 encoding *sulP-CS, sbt-CS*, *sbt, chr,* or *sulP,* in which codons were optimized for expression in *E. coli* (49). Plasmids were provided in *E. coli* Top 10 (Invitrogen). The nucleotide sequences encoding the two subunits of the DAC from *T. pelophila* were retrieved from the Integrated Microbial Genomes and Microbiomes Database (IMG gene ID numbers 2568511024 and 2568511025) and provided to GenScript, which synthesized and cloned them into pBAD.

A double CA knockout strain of *E. coli* [*E. coli* Lemo21(DE3) *ΔyadF ΔcynT* (20)] was selected as the host strain for all plasmids. Wild-type *E. coli* meets its requirements for HCO_3_^-^ for biosynthesis via CA; this double knockout requires elevated CO_2_ for growth and can be rescued via heterologous expression of DIC transporters or CA (20, 29). *E. coli* Lemo21(DE3) *ΔyadF ΔcynT* was made chemically competent via the CaCl_2_ method (20, 50). Plasmids were isolated from *E. coli* Top 10, using the QIAprep spin minikit (Qiagen) and transformed into chemically competent *E. coli* Lemo21(DE3) *ΔyadF ΔcynT*. Transporter gene expression was verified via LC-MS-MS as in (29).

Inocula for growth assays were prepared as in (29). Growth assays were performed under the influence of inducer (900 mg L^−1^ arabinose) or repressor (2 g L^−1^ glucose) and the appropriate antibiotics (30 mg L^−1^ apramycin, 30 mg L^−1^ chloramphenicol). Additionally, *E. coli* Lemo21(DE3) *ΔyadF ΔcynT*, which was not carrying a plasmid (no plasmid control), as well as a positive control (*E. coli* carrying DAC from *H. crunogenus*) was run alongside each experiment. Flasks were incubated with an air headspace at 37°C, 100 rpm, and growth was monitored via spectrophotometry at 600 nm for 30 h.

### Confirming DIC transport capabilities of potential DIC transporters

Silicone oil centrifugation was used to verify whether potential DIC transporters were capable of DIC transport (Fig. 1). The concentration of intracellular DIC in *E. coli* Lemo21(DE3) *ΔyadF ΔcynT* carrying the plasmids was measured via DI^14^C accumulation and silicone oil centrifugation, modified from (12) for use in *E. coli* as in (20). *E. coli* was cultivated under an air headspace in lysogeny broth supplemented with arabinose as described above. Plasmid-free *E. coli* Lemo21(DE3) *ΔyadF ΔcynT* were also cultivated, under 5% CO_2_, 95% air, to act as a negative control for DIC transport. Intracellular DIC concentrations were measured eight times per construct.

### Statistical analyses

Statistical analyses were implemented in R v.4.3.2 (51). For determining the response of µ to [DIC], for each of 15 chemostats, three measurements of µ and [DIC] were taken once they reached steady state. The mean values of µ and [DIC] from each chemostat were used to determine the K_DIC_ and µ_max_ by fitting the Monod equation to the data via nonlinear regression via the drc package, drm() function, using the MM.2() function to specify the Michaelis-Menten equation (identical to the Monod equation; [52]). The drm() function also calculated the standard errors of the parameters estimated from the fitted curve.

To estimate K_DIC_ and V_max_ values for whole cells and purified RubisCO, the Michaelis-Menten equation was fitted to the carbon fixation rates and [DIC] using the drc package drm() function as described above for chemostat data. For whole cells, these parameter values are accompanied by the standard errors estimated from the fitted curves. For RubisCO, since three independent measurements of DIC fixation rates were undertaken, standard deviations of the parameter values were calculated from the means of the three K_DIC_ and V_max_ values.

Chi-square tests were used to determine whether carboxysome counts differed between growth conditions (DIC-limited vs. NH_3_-limited) or species (*T. pelophila* vs. *H. crunogenus*). Since two chemostats were grown for each condition, tallies in each category (e.g., 0, 0.25, carboxysomes µm^2^) were averaged, and the average values were compared using the chisq.test() function in R.

In order to determine whether transcript abundances for each target gene differ between cells growing under DIC-limited vs NH_3_-limited conditions, qRT-PCR reactions for each gene (reference citrate synthase, and target CCM genes) were run in triplicate for each chemostat. Mean and standard deviations for each Ct value were calculated. △Ct values were calculated from these mean Ct values, and their standard deviations were calculated via error propagation from the standard deviations of the Ct values. In order to calculate △△Ct values, each DIC-limited chemostat was paired with an NH_3_-limited chemostat; △△Ct values were calculated from the △Ct values from each of the two chemostats, and standard deviations were calculated via error propagation. An average △△Ct value was calculated from all three pairs, and standard deviations were calculated via error propagation. In order to determine whether these △△Ct values differed from zero (no difference in transcript abundance), *t-*values were calculated from △△Ct values and their standard errors. Values of *P* were determined in R using the pt() function.

In order to determine whether potential DIC transporter genes elevated intracellular DIC concentrations when expressed in *E. coli,* intracellular DIC values for each construct were compared to those measured in a plasmid-free control using the t.test() function in R.

## RESULTS

### Growth of *T. pelophila* in chemostats and kinetic parameters for growth

**Fig 2:**
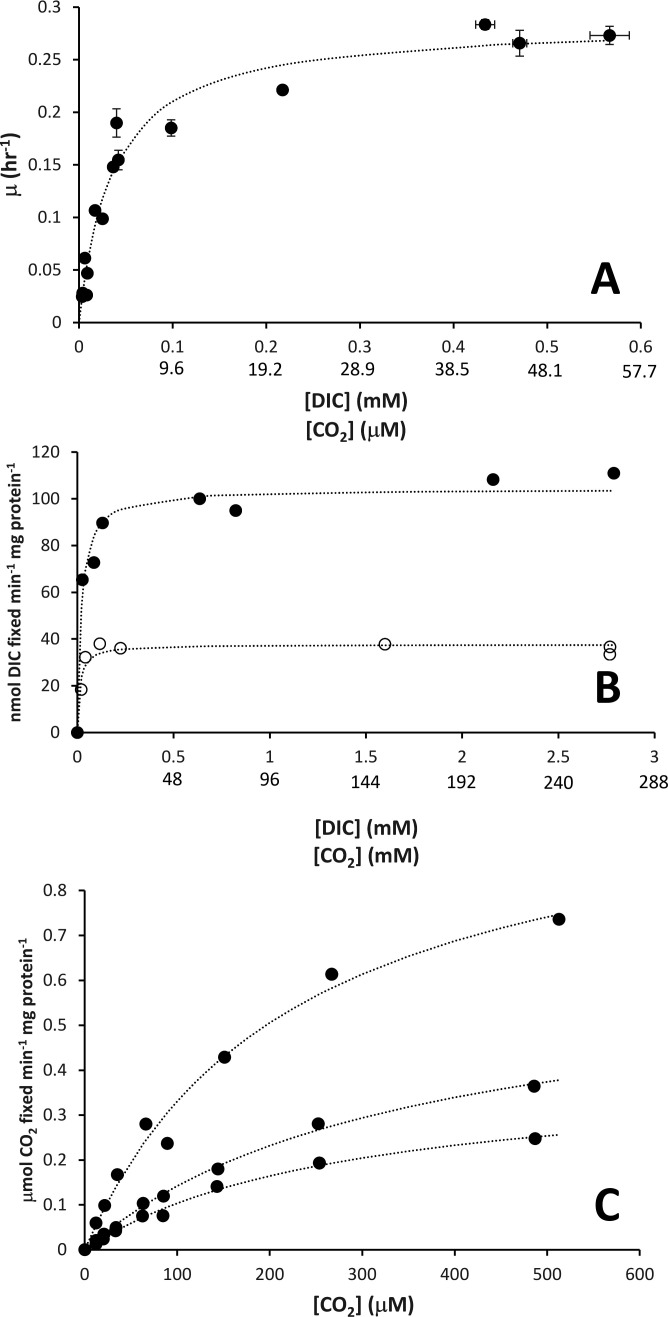
Response of *T. pelophila* growth rate constants (μ), whole-cell dissolved inorganic carbon (DIC) fixation rates, and RubisCO CO_2_ fixation rates to the concentration of DIC and CO_2_. (**A**) µ values and steady-state concentrations of DIC from chemostat growth chambers are plotted from 15 DIC-limited chemostats. (**B**) DIC fixation rates as a function of DIC concentrations from cells harvested from two different chemostats with µ = 0.05 hr^−1^. One chemostat was DIC-limited (and NH_3_-replete; open circles), while the other chemostat was NH_3_-limited (and DIC-replete; closed circles). In **A and B**, CO_2_ concentrations are calculated from the concentration of DIC as described in Materials and Methods. (**C**) Three independent determinations of CO_2_ fixation from purified RubisCO. In **A–C**, the dotted curves are based on the Monod (**A**) or Michaelis-Menten (**B, C**) equations fitted to the data.

*T. pelophila* was successfully cultivated in chemostats under DIC limitation, at a range of growth rate constants. Cells grew rapidly even at low DIC concentrations, with K_DIC_ = 35±5 µM and μ_max_ = 0.29±0.01 hr^−1^ (Fig. 2).

### Parameters for DIC fixation by cells and purified RubisCO

DIC fixation by *T. pelophila* cells harvested from DIC-limited chemostats had K_DIC_ = 13.1 ± 0.4 µM, and V_max_ = 37 ± 2 nmol DIC min^−1^ mg protein^−1^. For NH_3_-limited cells, K_DIC_ = 20.8 ± 0.6 µM and V_max_ = 104 ± 4 nmol DIC min^−1^ mg protein^−1^ (Fig. 2). For purified RubisCO (Fig. S3), K_DIC_ = 6,749 ± 1,440 µM, and V_max_ = 0.7 ± 0.3 nmol CO_2_ min^−1^ mg protein^−1^. K_CO2_ = 285 ± 60 µM (Fig. 2; calculated from K_DIC_ values as described in Materials and Methods).

### Enumeration of carboxysomes in DIC-limited and NH_3_-limited cells

**Fig 3:**
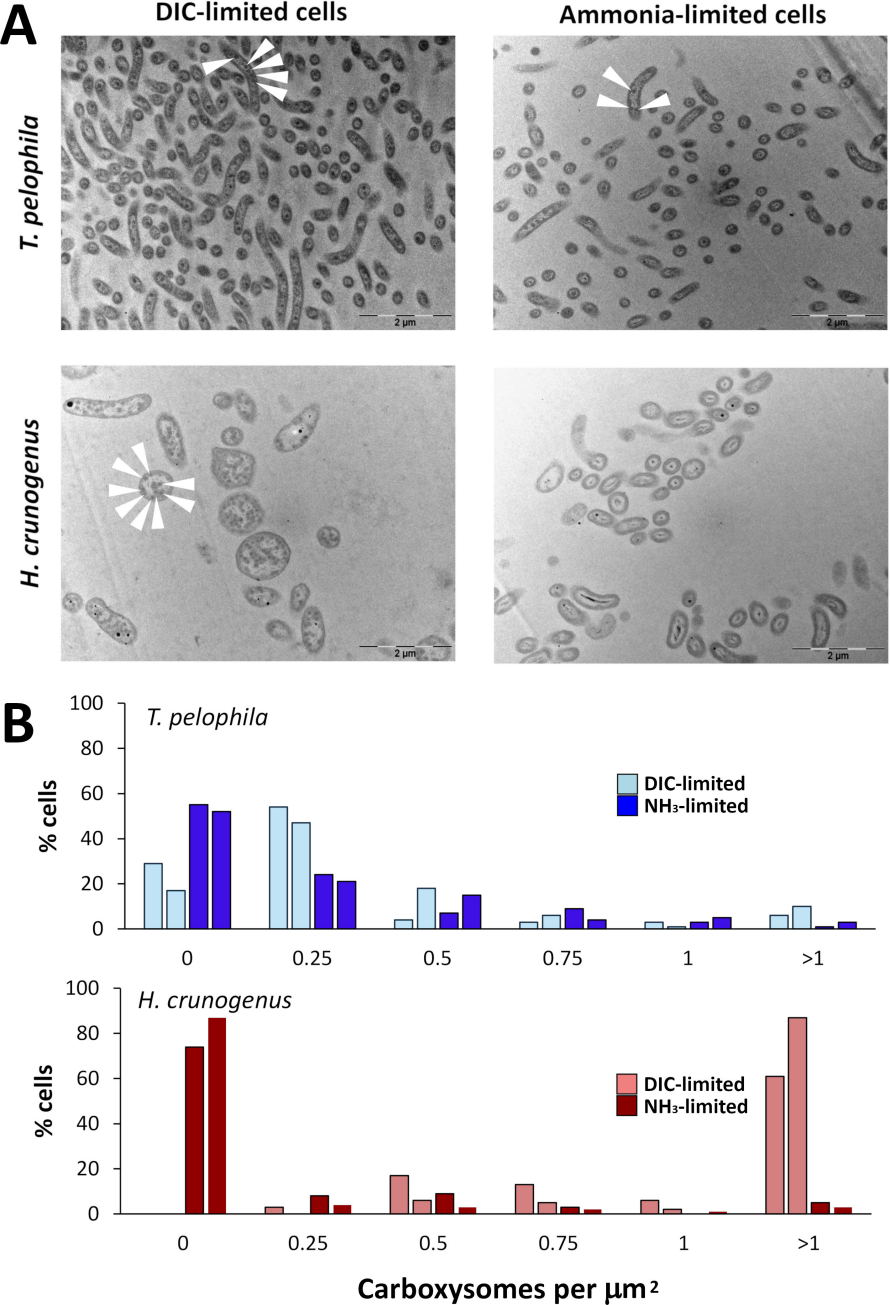
Carboxysome abundance in *T. pelophila* and *H. crunogenus* cultivated under DIC- or NH_3_-limiting conditions. (**A**) Transmission electron micrographs of thin sections of *T. pelophila* and *H. crunogenus*. White arrows indicate electron-opaque inclusions that were counted as carboxysomes, based on their round shape and diameter (100–200 nm). (**B**) Histograms from two chemostats per growth condition, depicting percentages of cells with a given number of carboxysomes per µm^2^ of cytoplasm transected in the electron micrographs.

Carboxysomes were enumerated from electron micrographs of *T. pelophila* and *H. crunogenus* grown under identical conditions of DIC or NH_3_ limitation (Fig. 3). Carboxysome abundances differed significantly in DIC vs. NH_3_-limited cells from both species (Table 1), and these differences were particularly pronounced for *H. crunogenus* (Fig. 3; Table 1) (*P*-value = 4.5e-5 [*T. pelophila*] vs. <2.2e-16 [*H. crunogenus*]). A difference between the species in their response to DIC limitation is also apparent from comparing the distribution of carboxysome abundances in DIC-limited *T. pelophila* vs. *H. crunogenus* (Table 1). The larger standardized residuals for the lowest (0) and highest (>1) bins when *H. crunogenus* cells cultivated under DIC limitation are compared either to *H. crunogenus* cells cultivated under NH_3_ limitation, or to *T. pelophila* cells cultivated under DIC limitation, indicate that a substantial portion of the differences between samples is due to differences in abundance of cells either with zero or >1 carboxysome per µm^2^. This trend is apparent in the histograms, in which *H. crunogenus* cells cultivated under low DIC conditions have carboxysome numbers that skew toward the largest bin included in the analysis (>1), and lack counts in the smallest bin (0).

**TABLE 1:** χ-square test for differences in carboxysome abundance in *T. pelophila* and *H. crunogenus* cells cultivated under DIC or NH_3_ limitation

Comparison	χ-square	*P*-value	Standardized residuals by bin^*a*^					
			0	0.25	0.5	0.75	1	>1
*T. pelophila* DIC-lim. vs. NH_3_-lim.	27.53	4.5e-5	−2.45, 2.45	2.34,−2.33	0.01,−0.01	−0.42, 0.41	−0.57, 0.57	1.34, −1.34
*H. crunogenus* DIC-lim. vs. NH_3_-lim.	154.14	<2.2e-16	−6.35, 6.37	−1.17, 1.17	0.92, −0.92	1.35, −1.35	1.16, −1.16	5.58, −5.60
DIC-lim.: *T. pelophila* vs. *H. crunogenus*	124.47	<2.2e-16	3.42, −3.40	4.84,−4.82	−0.06, 0.06	−0.86, 0.85	−0.57, 0.57	−5.13, 5.11
NH_3_-lim.: *T. pelophila* vs. *H. crunogenus*	21.63	6.1e-4	−1.65, 1.65	2.19, −2.19	0.86, −0.86	0.94, −0.94	1.17, −1.17	−0.58, 0.58

^*a*^ For a particular comparison (e.g., 1 vs. 2), standardized residuals are presented as 1, 2.

### Transcript abundances from genes encoding potential CCM components

Transcript abundances for genes encoding potential CCM components (carboxysomal shell proteins, CA, potential DIC transporters) were compared for *T. pelophila* cultivated under DIC or NH_3_ limitation as well as *H. crunogenus*, to act as a positive control (43). Fold changes in transcripts for most of the potential CCM-associated genes of *T. pelophila* were small, ranging from 0.4 to 1 (Table 2). Genes *dacM, dacC,* and *cbbM* had differences in transcript abundance (0.1–0.2), suggesting some downregulation under DIC limitation. One gene encoding a potential DIC-transporter (*sbt*) was dramatically upregulated under DIC limitation. In comparison, *dacM, dacC, cbbL* (carboxysomal), and *csoS1–3* in *H. crunogenus* showed a fold change of >70, under DIC-limiting conditions. Noncarboxysomal *cbbL* is upregulated under NH_3_-limiting conditions. Transcript response in *H. crunogenus* is consistent with prior measurements (31, 53), with the exception of *cbbM,* which is upregulated under DIC-limited conditions here and downregulated in a prior study using microarrays (31).

**TABLE 2:** Differences in transcript abundance for *T. pelophila* and *H. crunogenus* cultivated under low vs. high DIC conditions

Gene	Function	*T. pelophila* △△Ct value ±SE (*n* = 3)^*a*^	Fold difference^*b*^	*H. crunogenus* △△Ct value ±SE (*n* = 3)^*a*^	Fold difference^*b*^
Potential DIC transporter					
*chr*	Chromate ion transporter	0.21±0.80	0.8	0.04±0.59	1
*dacM*	Membrane subunit of DAC	2.66±0.86*	0.2	−7.31±0.71**	158
*dacC*	Cytoplasmic subunit of DAC	2.27±0.48*	0.2	−7.50±0.76**	180
*sbtA*	Sodium-dependent bicarbonate transporter	−8.13±0.42**	280	N/A*^c^*	N/A
*sulP*	Sulfate permease family transporter	1.06±0.85	0.5	−1.02±0.58	2
*sbtA-CS*	Sodium-dependent bicarbonate transporter encoded near carboxysome locus	1.25±0.83	0.4	N/A	N/A
*sulP-CS*	Sulfate permease family transporter encoded near carboxysome locus	−0.10±1.29	1	N/A	N/A
Carboxysome associated					
*cbbL-CS*	Large subunit of carboxysomal form I RubisCO	1.34±0.91	0.4	−6.57±0.71**	95
*csoS1*	Carboxysome shell protein	0.98±0.91	0.5	−6.24±0.74**	87
*csoS2*	Carboxysome assembly protein	1.37±0.83	0.4	−6.63±0.79**	99
*csoSCA*	CsoSCA-type carboxysomal CA	N/A	N/A	−7.47±0.71**	77
*ɩ–CA*	Iota-type carboxysomal CA	−0.26±0.23	1	N/A	N/A
*csoSX*	Hypothetical protein of unknown function	0.11±0.85	0.9	N/A	N/A
Other					
α-CA	Alpha CA	1.43±0.78	0.4	0.89±0.54	0.5
β-CA	Beta CA	N/A	N/A	−0.35±0.64	1
*cbbL*	Large subunit of noncarboxysomal form I RubisCO	N/A	N/A	7.97±0.64**	0.004
*cbbM*	Form II RubisCO	3.34±0.04**	0.1	−3.52±0.55*	12

^*a*^ Values significantly different from 0: *, *P* < 0.05; **, *P* < 0.01.

^*b*^ Fold difference = DIC limited / NH_3_ limited.

^*c*^ N/A, not applicable.

### Heterologous expression of potential DIC transporters

**Fig 4:**
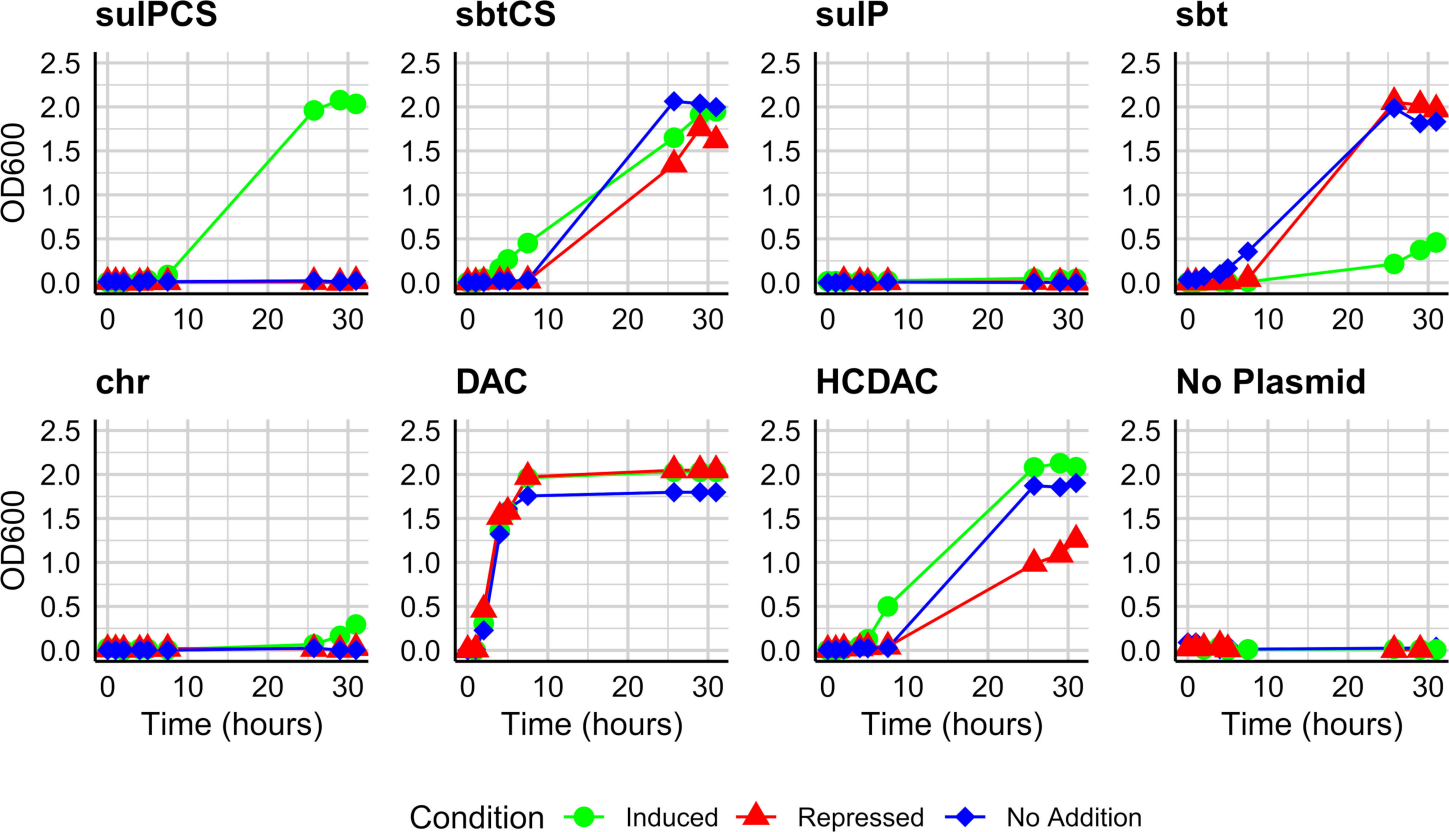
Growth of *E. coli* Lemo21(DE3) *ΔyadF ΔcynT* expressing potential DIC transporter genes from *T. pelophila* under low-CO_2_ conditions (0.04%-CO_2_). Each plot represents growth of *E. coli* expressing a different potential DIC-transporter: *sulPCS* – sulfate permease family adjacent to the carboxysome locus; *sbtCS* – sodium-dependent HCO_3_^-^ transporter adjacent to the carboxysome locus; *sulP -* sulfate permease family; *sbt* – sodium-dependent HCO_3_^-^ transporter; *chr* – chromate ion transporter family; *DAC* – multi-subunit transporter; *HCDAC – E. coli* carrying a plasmid encoding the DAC accumulating complex from *H. crunogenus;* served as a positive control. No plasmid – *E. coli* without a plasmid; served as a negative control.

Potential DIC transporters were expressed in *E. coli* (Table S2). Expression of SulP-CS, Sbt-CS, Sbt, and DAC from *T. pelophila* rescued *E. coli* [Lemo21(DE3) *ΔyadF ΔcynT*] from its requirement for high CO_2_ concentrations for growth, enabling this strain of *E. coli* to grow under low-CO_2_ conditions (Fig. 4). For SulP-CS, Sbt-CS, and Sbt, the presence of arabinose (inducer) stimulated growth; longer lag phases were apparent when glucose (repressor) was added. For DAC, similar growth was observed in the presence of inducer or repressor, and this complex was detected in cells cultivated under both conditions (Table S2). SulP and Chr were unable to rescue growth by this strain of *E. coli,* suggesting that they may not be capable of DIC transport*.* It is also possible that they misfolded when expressed in *E. coli*.

### Intracellular DIC accumulation in *E. coli* expressing potential DIC transporters

**Fig 5:**
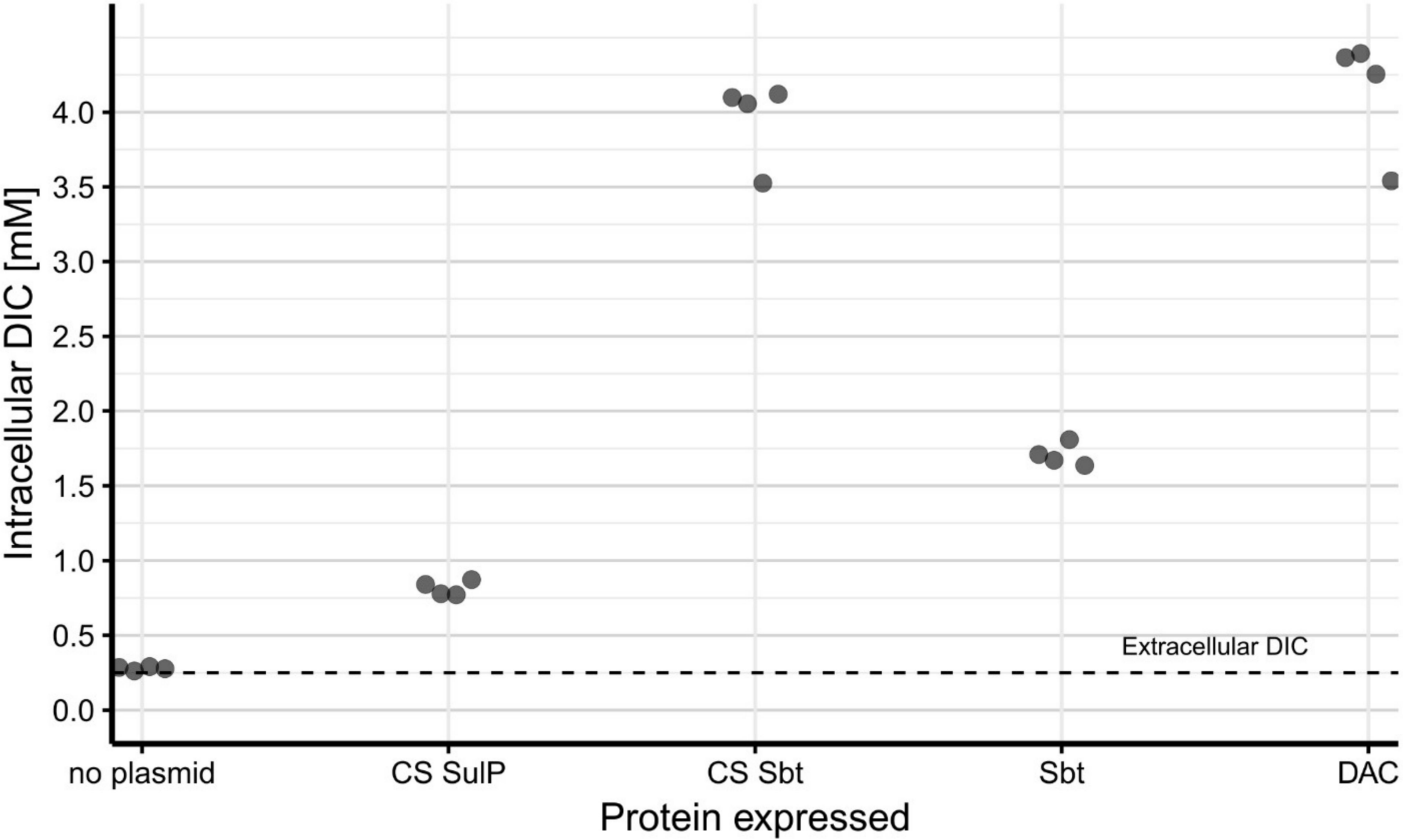
Intracellular DIC concentrations measured in *E. coli* Lemo21(DE3) *ΔyadF ΔcynT* expressing potential DIC transporter genes from *T. pelophila.* No plasmid – negative control; CS SulP – sulfate permease family adjacent to the carboxysome locus; CS Sbt – sodium-dependent HCO_3_^-^ transporter adjacent to the carboxysome locus; Sbt – sodium-dependent HCO_3_^-^ transporter; DAC – multi-subunit transporter. The extracellular DIC concentration was 0.25 mM and is shown as a dashed line for comparison.

All transporters that rescued *E. coli* Lemo21(DE3) *ΔyadF ΔcynT* were able to generate measurably elevated intracellular DIC concentrations in their absence (Fig. 5, *P* < 0.05).

## DISCUSSION

### Similarities and differences between the CCM of *T. pelophila* and other bacteria

It can be concluded that *T. pelophila* does encode and express a functional CCM, based on multiple lines of evidence presented here. *T. pelophila* can grow under very low DIC conditions (Fig. 2)*.* Carboxysomes are visible in EMs (Fig. 3) and contain active RubisCO (Fig. 2). The K_CO2_ of this RubisCO (285 µM) is far larger than whole-cell K_CO2_ values from the response of µ to DIC (K_CO2_ = 2 µM), and the response of DIC fixation rate to DIC concentration in cells grown under low (K_CO2_ = 1.2 µM) or high (K_CO2_ = 2.0 µM) DIC (Fig. 2).

The CCM in *T. pelophila* has characteristics that distinguish it from others. Most notably, carboxysome transcript abundance is similar under low and high DIC conditions (DIC-limited or NH_3_-limited, respectively), and carboxysome abundance differs only modestly, which is consistent with an earlier study that measured a lack of impact of DIC availability on RubisCO activities in this organism (33). Other *Gammaproteobacteria* with CCMs, such as members of the genus *Thiomicrorhabdus* and *Hydrogenovibrio*, have dramatically elevated numbers of carboxysomes and corresponding transcript abundances when they are cultivated under low DIC conditions (19, 43). To confirm this peculiar result, *H. crunogenus* was cultivated here under conditions identical to those used for *T. pelophila*, and as before, it had dramatically elevated carboxysome numbers and associated transcripts (Tables 1 and 2; Fig. 3). *T. pelophila* is more similar in this regard to what has been observed in *Cyanobacteria,* which constitutively express carboxysomes and have modest (~2 fold) increases in carboxysome numbers when grown under low DIC conditions (54, 55). The dramatic upregulation of carboxysome genes observed in other sulfur-oxidizing chemolithoautotrophs is energetically expensive; low DIC conditions can result in dozens of carboxysomes apparent per cross-sectional cell slice (Fig. 3) (19). It is likely that a substantial portion of protein synthesis is allocated to carboxysome synthesis under these conditions. Perhaps the less dramatic changes in carboxysome numbers apparent in *T. pelophila* represent a strategy to avoid the enormous energy allocation by other chemolithoautotrophs to carboxysome synthesis under low DIC conditions. Many of the organisms with dramatic changes were originally isolated from hydrothermal vent environments (19, 56). It is possible that such an energetic expense could be maladaptive in a non-hydrothermal coastal marine sediment environment, such as the habitat from which *T. pelophila* was originally isolated (34), in which redox substrates are delivered via diffusion, than in a hydrothermal vent environment, where they can be delivered more rapidly by bulk fluid flow (57).

### *T. pelophia* DIC-transporters are regulated differently from those of other bacteria utilizing CCMs

Other organisms with CCMs upregulate multiple DIC transporters when exposed to low DIC conditions. For example, *Hydrogenovibrio thermophilus JR2* upregulates all three of its DIC transporters in response to DIC limitation (19). Genes encoding four DIC transporters are present in the *T. pelophila* genome; however, only one of these transporters (Sbt) was upregulated here under DIC limitation (Table 2). There are three possible explanations for this absence of upregulation for the other three DIC transporters: (i) these transporter genes are constitutively expressed under the conditions used in this study, (ii) these three transporters are not expressed under the conditions of this study, or (iii) either of the former is true, but the genes respond to conditions beyond those used in this study. The high affinity of cells for CO_2_ and the relatively small changes in whole cell K_DIC_ (13–21 µM) measured here are consistent with the first possibility. The third possibility is supported by an earlier study using a different growth medium (TASW, pH 7.5), in which the multi-subunit DIC transporter DAC was upregulated by 100-fold, when comparing *T. pelophila* cultivated under DIC vs. ammonia limitation (19). It is possible that differences in pH, DIC, and ionic composition, or comparison with phosphate or thiosulfate-limited cells instead of NH_3_-limited cells, could result in their differential transcription. In contrast, DAC was upregulated in *H. crunogenus* whether cultivated in TP or TASW medium (12) (Fig. 3; Table 2).

While differing from CCMs from chemolithoautotrophs whose CCMs have been characterized, it is important to recognize that placing the CCM from *T. pelophila* in a broader taxonomic/evolutionary context is compromised by the relative paucity of well-studied chemolithoautotrophic CCMs. Among members of phylum *Pseudomonadota,* only three members (all *Gammaproteobacteria)* have well-characterized CCMs (e.g., chemostat studies; ultrastructure studies; carboxysome characterization; Measurement of intracellular DIC accumulation; DIC transporter verification): *Ha. neapolitanus* (order *Chromatiales*), *T. pelophila,* and *H. crunogenus* (order *Thiotrichales*) (9, 11, 12, 29, 43). Other members of *Thiotrichales,* and one member of the order *Acidithiobacilliales*, have been shown to be likely to have CCMs based on their ability to grow under low DIC conditions, during which they upregulate genes encoding carboxysome components and likely DIC transporters (19, 58). The presence of carboxysome loci and genes encoding likely DIC transporters in at least 10 orders from *Alpha-, Beta-,* and *Gammaproteobacteria* strongly suggests that CCMs are present among a taxonomically and physiologically broad group of microorganisms (59). Perhaps some of these organisms employ a CCM strategy similar to the one observed in *T. pelophila.*

### The presence of multiple DIC transporters could offer a selective advantage in alkaline environments

Genomes from members of the genus *Thiomicrospira* encode 3–5 DIC potential transporters, while genomes of genera *Hydrogenovibrio* and *Thiomicrorhabdus* encode 1–3 (19). Perhaps this functional redundancy makes their CCMs more robust. In an earlier study, a random knockout library of 10,000 T*. pelophila* mutants were generated by transposon-mediated random mutagenesis (29). While the interruption of carboxysome genes led to CO_2_-sensitive *T. pelophila* mutants, no CO_2_-sensitive mutants with disrupted DIC transporter genes were detected (29). Mutants with a single interrupted DIC transporter were likely still able to grow under low DIC conditions by upregulating one of the other DIC transporter genes. It would be of great interest to observe the phenotypes of mutant strains of *T. pelophila* in which each of the DIC transporter genes was knocked out. Such studies await the generation of a system to successfully introduce site-directed mutations in this organism.

The presence of more genes encoding DIC transporters may be an adaptation to growth in high pH environments, since CO_2_ will always be scarce in high pH environments, and active transport of HCO_3_^-^ will always be necessary for growth. Most members of the genus *Thiomocrospira* have been isolated from extremely alkaline environments, mainly soda lakes, with optimal pH values for growth ranging from 9.2 to 11 (60). These different transporters could be regulated differentially based on environmental conditions. For example, since Sbt transporters have been found to have a higher affinity for HCO_3_^-^, these could be differentially expressed when DIC is particularly low, as seen in *Cyanobacteria* (16). Both SulP and Sbt transporters require sodium to transport HCO_3_^-^ (21) and may have different affinities for this ion, which may create conditions under which one transporter would be more effective than the other. Unlike the alkaliphilic members of its genus (*T. aerophila, T. microaerophila, T. cyclica, T. ALE5*), the genomes of neutrophilic *T. pelophila* and *T. thyasirae* also encode DACs, which transport CO_2_ (20). DAC-type transporters could provide an advantage for DIC transport at neutral pH, where CO_2_ is also present.

Active DIC transport may provide an advantage in an industrial context as well. Currently, synthetic biology is being used to assemble autotrophic pathways in a variety of organisms to synthesize precursor compounds from CO_2_ (61–64). CCM components could enhance these pathways by speeding the supply of CO_2_ and HCO_3_^-^ to the cytoplasm. Examining CCMs that originate from a variety of physiological, taxonomic, and environmental contexts will help in the design of organisms that can function in a variety of industrial contexts, especially under variable or low DIC conditions, or at alkaline pH. The CCM observed in this study was most likely accrued from extreme alkaliphilic ancestors and could provide clues to construct CCMs that function in industrial settings in which the pH is not neutral. Furthermore, an understanding of CCMs has relevance beyond autotrophy; DIC metabolism is essential to the biosynthesis of nucleobases, amino acids, and fatty acids by all organisms that synthesize these building blocks (reviewed in [65]). Indeed, genes encoding likely DIC transporters and CAs are common in all genomes (65), and the presence of DIC transporters has been found to enhance growth and virulence in *Staphylococcus aureus* (66). Including CCM components in heterotrophic organisms used for industrial processes could enhance production of target compounds by these organisms as well.

## Data Availability

Nucleotide sequences of transporter genes optimized for expression in *E. coli* are available at GenBank (accession numbers PV963820 to PV963826).

## SUPPLEMENTAL MATERIAL

Supplemental material aem.01529-25-s0001.docx Tables S1 to S3; Fig. S1 to S3.

ASM does not own the copyrights to Supplemental Material that may be linked to, or accessed through, an article. The authors have granted ASM a non-exclusive, world-wide license to publish the Supplemental Material files. Please contact the corresponding author directly for reuse.
