# Supplementary material for: Unusual regulation of the CO2 concentrating mechanism of marine chemolithoautotroph Thiomicrospira pelophila
Source: Appl Environ Microbiol. 2025 Oct 20;91(11):e01529-25. doi: 10.1128/aem.01529-25 (PMC12628692; doi:10.1128/aem.01529-25)
Supplement: Supplemental material — Tables S1 to S3; Fig. S1 to S3. [file aem.01529-25-s0001.docx]

**TABLE S1.** Primers used for qRT-PCR

| **Taxon** | **IMG gene object ID^a^** | **Predicted gene product^b^** | **Forward primer** | **Reverse primer** |
| --- | --- | --- | --- | --- |
| *T. pelophila* | 2568509996 | CbbL (CS) | AAGTTATTCCACAAGCTGGCG | TCAACAGGTCAGTCCATACAGTT |
|  | 2568510003 | CsoS1 | TGGTGCTGATGCTTGTGAG | GCTGGGTTAATACCTGCAGTTAAA |
|  |  |  |  |  |
|  | 2568510028 | CbbM | CGGGTTATGGTTACCTAGAAGTG | CATCAATCTCATAAACCAATGCGTC |
|  | 2568511177 | α CA | GAACAAGGCCAAGTGCAAAC | GTTTTGTGTCAGATTTATTAGCAC |
|  | 2568510010 | ι CA (CS) | TAACGGGTGTTTCATCT | GCCGCATTCCATTTATC |
|  |  |  |  |  |
| *H. crunogenus* | 637785558 | CbbL (CS) | ATGGCAAGTAAAACGTTTGATGC | TGAAACAAGCCAATAGATCGGTATC |
|  | 637785565 | CsoS1 | AGTACGGAATATGGAATTGCATTAGG | GAACTTCAGCTGCTTTAGTCATAGC |
|  | 637785560 | CsoS2 | AGAGACAGCAACAGAAGAACG | CAATCGGCTCTTCAGGTTTTTG |
|  | 637785138 | CbbL | ATGGCTAAGACTTATAACGCCG | GTGGAACACCATCCTGTGG |
|  | 637785135 | CbbM | ATGGATCAGTCGAATCGTTATGC | CAGGTTCCATTGTGTAAGCAAC |
|  | 637786281 | α CA | GCCGCACCATTGATTGATTTAG | TAGTGGCGGACTCTTTTTCAG |
|  | 637785132 | β CA | ATGTGCCATCAATGTGACTGC | GGACGAATGGCGTTGATTTC |
|  |  |  |  |  |

^a^Gene object identification numbers are from the Integrated Microbial Genomes system (https://img.jgi.doe.gov)

^b^Gene product abbreviations: CbbL: Large subunit of RubisCo; CbbM: Form II RubisCo; CsoS1: Carboxysomal shell protein; CsoS2: Carboxysome assembly protein; α CA: Alpha carbonic anhydrase; β CA: beta carbonic anhydrase; ι CA (CS): Iota carbonic anhydrase. Gene product names followed by (CS) indicate that these genes are adjacent to those encoding the components of carboxysomes.

**TABLE S2**. Verification of *T. pelophila* transporter expression in *E. coli* via LC-MS-MS

| **Target** | **Intensity of target in sample when induced** | **Intensity of target in sample when repressed** | **Intensity of target in no-plasmid control** | **Unique peptides identified from target** | | **Target sequence coverage [%]** |
| --- | --- | --- | --- | --- | --- | --- |
| CS-SulP^1^ | 1.57E+08 | NM^2^ | ND^3^ | 3 | | 7 |
| CS-Sbt^1^ | 8.26E+07 | NM | ND | 1 | | 3.3 |
| SulP | 1.77E+09 | NM | ND | | 1 | 22.1 |
| Sbt | 9.34E+06 | NM | ND | 2 | | 11 |
| DAC-C^5^ | 4.55E+10 | 1.91E+09 | ND | 46 | | 75.1 (I)^4^, 73.4 (R)^4^ |
| DAC-M^5^ | 1.41E+09 | 8.92E+07 | ND | 11 | | 16.6 (I), 8.1 (R) |
| Chr | 3.13E+08 | NM | ND | 2 | | 7.5 |

^1^’CS’ indicates that the gene encoding this transporter is collocated with the carboxysome locus in the genome

^2^Not measured

^3^Not detected

^4^I - induced, R - repressed

^5^In *T. pelophila,* the DAC consists of cytoplasmic (C) and membrane-spanning (M) subunits

**TABLE S3**. Growth conditions tested to optimize *T. pelophila* growth medium

| **Condition tested** | **pH** | **Headspace**  **(% v/v O_2_, balance N_2_)** | **(NH_4_)_2_SO_4_ (g L^-1^)** | **Na_2_S_2_O_3_ (mM)** |
| --- | --- | --- | --- | --- |
| pH | 6, 6.5, 7, 7.5, 8 | 21 | 1 | 40 |
| Oxygen | 7 | 0, 0.2, 1, 5, 21, 100 | 1 | 40 |
| (NH_4_)_2_SO_4_ | 7 | 21 | 0, 0.01, 0.05, 0.1, 0.5, 1.0 | 40 |
| Na_2_S_2_O_3_ | 7 | 21 | 1 | 0, 5, 10, 20, 40 |


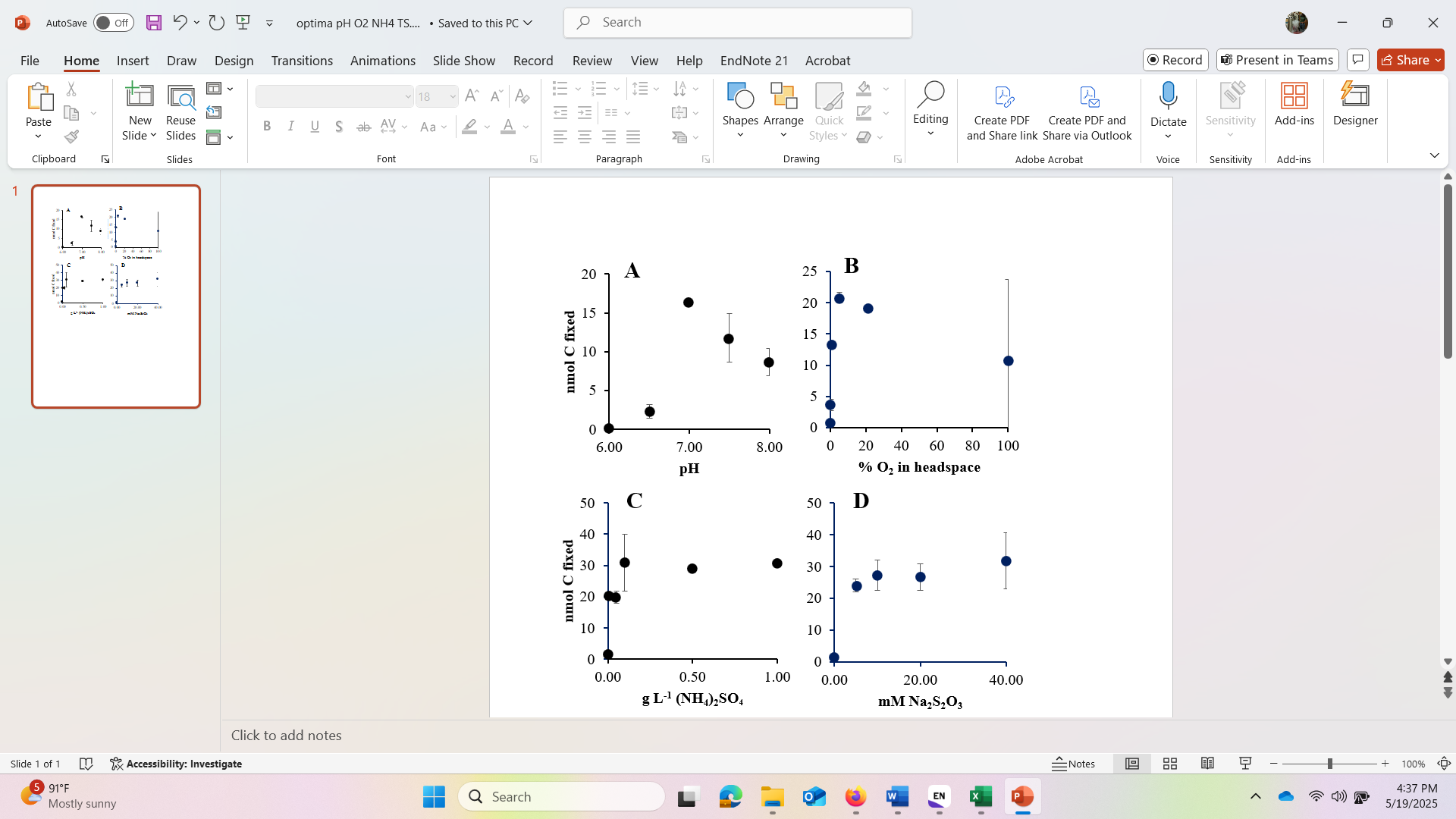


**FIG S1** Response of *T. pelophila* growth to **A)** pH, **B)** O_2_, **C)** (NH_4_)_2_SO_4_, and **D)** Na_2_S_2_O_3_ (thiosulfate). These responses were gathered to determine the best conditions for cultivating *T. pelophila* for the experiments of this study. Initial experiments used optical densities to track growth. While it was clear that growth had reached stationary phase 3-5 days after inoculation, optical densities included the elemental sulfur produced by this organism, and the experiments were repeated by assaying growth by DI^14^C assimilation. Growth medium was supplemented with 1 μCi NaH^14^CO_3_. 20 ml portions were amended and sealed under headspaces as described in Table S3. 50 μl of a turbid overnight culture was injected into each serum vial, and agitated (100 rpm) at 20°C for 5 days. Three portions of 0.2 ml were pipetted into scintillation vials containing 0.2 ml glacial acetic acid, vortexed, and allowed to degas overnight to remove any remaining NaH^14^CO_3_. To measure assimilated DI^14^C, 5 ml scintillation fluid (Scintiverse SX; Fisher Scientific) was added and samples, as well as 0.01 ml portions removed before acidification (for initial activity measurements), were analyzed with a scintillation counter (1).


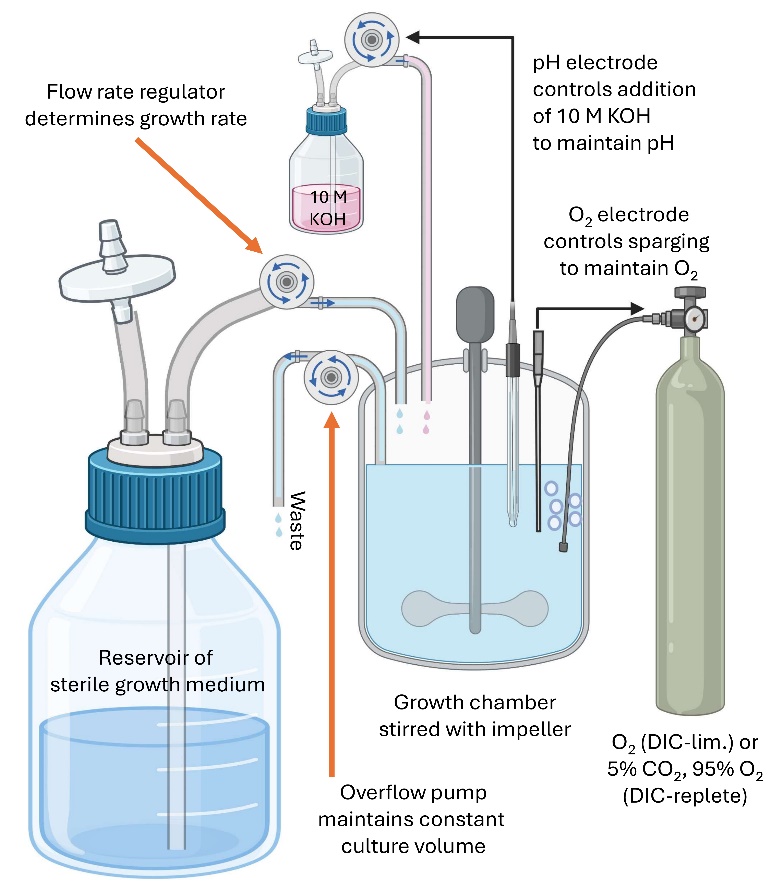


**FIG S2** Schematic of the chemostats used in this study. Growth media in the reservoir were designed to generate either DIC-limited, ammonia-replete or DIC-replete, ammonia-limited comditions in the growth chamber as the cells grew, as in the Methods and (1). Image created in BioRender. Scott, K. (2025) <https://BioRender.com/tkzf3yy>.


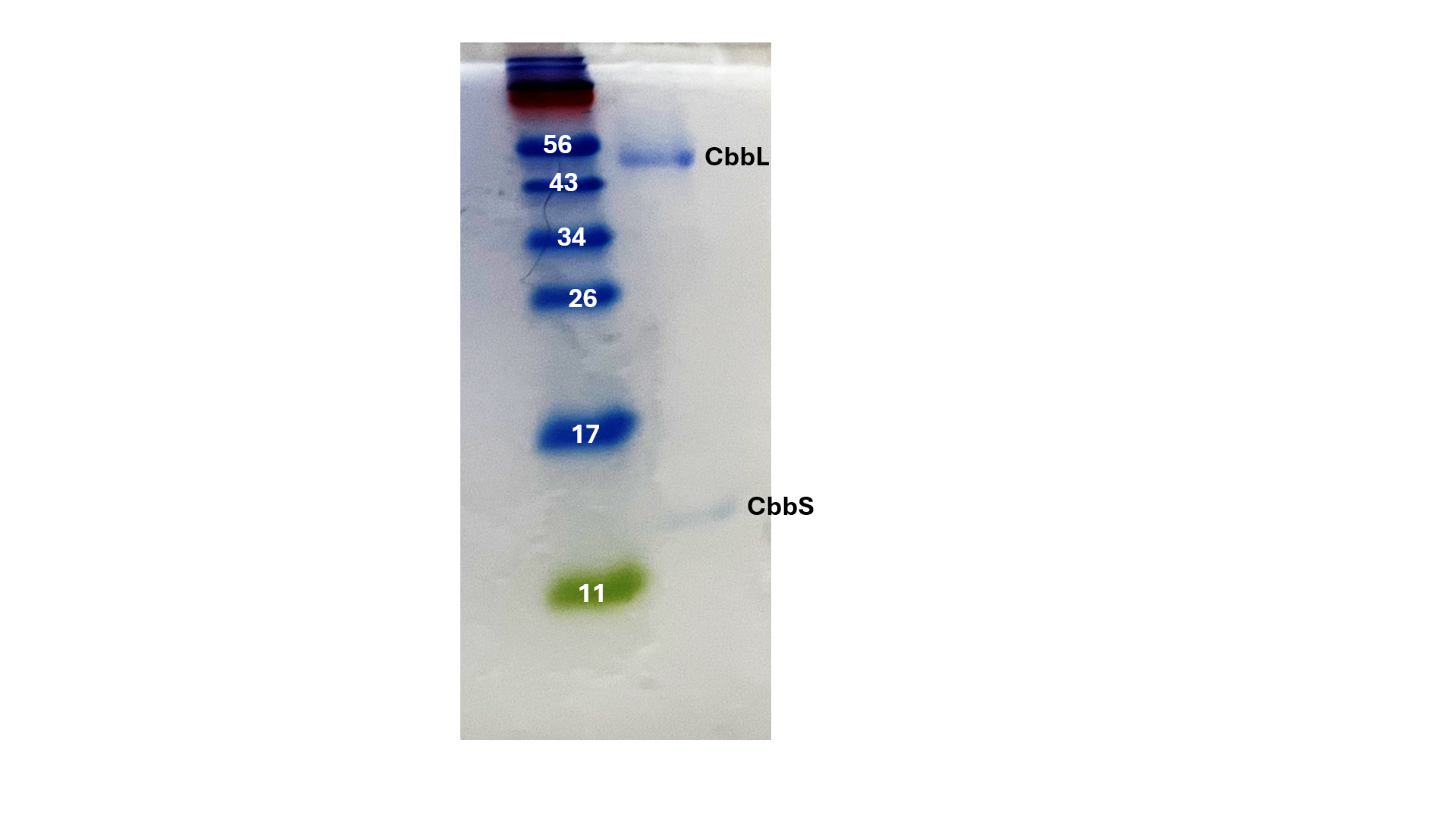


**FIG S3** SDS-PAGE analysis of RubisCO purified from *T. pelophila* carboxysomes as in (2). 20 μg of purified RubisCO was loaded on the gel, and two bands are visible, corresponding in size to the large (CbbL; 51.9 kDa) and small (CbbS; 13.3 kDa) subunits of this enzyme The protein ladder is labelled in kDa.

1. Dobrinski KP, Longo DL, Scott KM. 2005. A hydrothermal vent chemolithoautotroph with a carbon concentrating mechanism. J Bacteriol 187:5761-5766.

2. Menning KJ, Menon BB, Fox G, Scott UMLKM. 2016. Dissolved inorganic carbon uptake in *Thiomicrospira crunogena* XCL-2 is Dp- and ATP-sensitive and enhances RubisCO-mediated carbon fixation. Arch Microbiol 198:149-159.
